# Area based stratified random sampling using geospatial technology in a community-based survey

**DOI:** 10.1186/s12889-020-09793-0

**Published:** 2020-11-10

**Authors:** Carrie R. Howell, Wei Su, Ariann F. Nassel, April A. Agne, Andrea L. Cherrington

**Affiliations:** 1 Department of Medicine, Division of Preventive Medicine, University of Alabama at Birmingham, Medical Towers 62, 1717 11th Avenue South, Birmingham, AL 35205 USA; 2 School of Public Health, University of Alabama at Birmingham, 1665 University Blvd, Birmingham, AL 35233 USA

**Keywords:** Area based, Geographic information systems, Stratified random sampling, Hispanic population, Rural population, Community based methods

## Abstract

**Background:**

Most studies among Hispanics have focused on individual risk factors of obesity, with less attention on interpersonal, community and environmental determinants. Conducting community based surveys to study these determinants must ensure representativeness of disparate populations. We describe the use of a novel Geographic Information System (GIS)-based population based sampling to minimize selection bias in a rural community based study.

**Methods:**

We conducted a community based survey to collect and examine social determinants of health and their association with obesity prevalence among a sample of Hispanics and non-Hispanic whites living in a rural community in the Southeastern United States. To ensure a balanced sample of both ethnic groups, we designed an area stratified random sampling procedure involving three stages: (1) division of the sampling area into non-overlapping strata based on Hispanic household proportion using GIS software; (2) random selection of the designated number of Census blocks from each stratum; and (3) random selection of the designated number of housing units (i.e., survey participants) from each Census block.

**Results:**

The proposed sample included 109 Hispanic and 107 non-Hispanic participants to be recruited from 44 Census blocks. The final sample included 106 Hispanic and 111 non-Hispanic participants. The proportion of Hispanic surveys completed per strata matched our proposed distribution: 7% for strata 1, 30% for strata 2, 58% for strata 3 and 83% for strata 4.

**Conclusion:**

Utilizing a standardized area based randomized sampling approach allowed us to successfully recruit an ethnically balanced sample while conducting door to door surveys in a rural, community based study. The integration of area based randomized sampling using tools such as GIS in future community-based research should be considered, particularly when trying to reach disparate populations.

## Background

Obesity is a leading risk factor for the development of diabetes, cardiovascular illness, cancer and other chronic conditions that cause significant morbidity and mortality as well as increased health care costs [1]. Hispanics are the largest and fastest growing racial/ethnic minority group in the United States, comprising 17.3% of the population in 2014 [2], with disproportionately high obesity rates. Among adults living in the United States in 2015, the prevalence of obesity was 47% among Hispanics compared to 38% among non-Hispanic whites [3], highlighting the need to examine factors that contribute to this increased risk. To date, most studies among Hispanics have focused on individual risk factors of obesity, with less attention on interpersonal, community and environmental determinants. In order to conduct community level surveys to collect this type of data, it is crucial to ensure representativeness of both Hispanic and non-Hispanic populations in the study sample. Here we describe the use of a novel GIS-based population based sampling approach to minimize selection bias in a community based study.

Sampling for cross-sectional survey studies can be probability based or non-probability based. Probability based (e.g. random sampling) requires a defined population, where each possible unit has a known possibility of being selected [4]. Non-probability sampling methods (e.g. convenience sampling) have no known inclusion probabilities [5], producing bias and unbalanced sample representation [6–14]. Simple random sampling can also pose a problem for studies conducting research in minority populations. This method targets the whole population of interest and often results in minority under-representation. Stratified random sampling increases sample representativeness by dividing the study population into strata based on characteristics that are of interest to the researcher [15]. Random samples are then drawn from each strata to ensure adequate sampling of all groups. This approach reduces sampling bias; allows researchers to estimate within and between strata outcomes; and improves accuracy of results [15, 16].

Sampling design is important in large population studies with several national surveys utilizing stratified approaches to minimize bias. The US Census Bureau conducts the American Community Survey (ACS) to produce annually updated census data estimates based on geographic units (e.g. census tract and block group). The complex sampling design consists of first stratifying the US population by census block, then calculating population based sampling rates. Appropriate weights are applied in the analytical phase so that estimates represent the full population [17]. Similarly, the National Health and Nutrition Examination Survey (NHANES) employs a stratified, multistage cluster design that oversamples specific subgroups to increase precision in health outcome estimates [18]. Smaller scale community based population studies should draw upon and incorporate aspects of these rigorous sampling designs to reduce sampling error and increase precision in estimates.

In recent years, technologies such as Geographic Information System (GIS) have been used to facilitate the sampling process in community-based research. Typically, GIS software have been used for data analysis and visualization [19]; however, health researchers have begun to realize its potential in facilitating the sampling and recruitment process, particularly in rural, developing countries [20–22]. To aid in sampling, GIS has been used to define populations in areas without formal census data [21, 22]; create clusters [22]; and stratify populations [20]. Area stratified random sampling methods use area units as the strata, such as census blocks, and produce samples comparable to random digit dialing recruitment approaches [20, 23, 24]. This method provides an innovative way to conduct community-based health survey research, particularly when the study area is small in population. Blending aspects of complex sampling design, such as those used in national surveys, with GIS methods has the potential to strengthen community based research. Here, we describe how geospatial data and Geographic Information Systems (GIS) were used to develop an area stratified random sampling protocol that ensured demographic balance in conducting a community-based, interviewer administered survey. The study’s main aim was to examine social determinants of health and their association with obesity prevalence among a sample of Hispanics and non-Hispanic whites living in a rural community in the Southeastern United States.

## Methods

### Participants and setting

The population of interest resided in Albertville, Alabama where researchers had previously conducted a cervical screening study aimed at Hispanic women [25]. Located in Marshall County in the northeastern side of the state, Albertville has a population of 21,160 with 64.7% non-Hispanic white and 30.2% Hispanic as of the 2010 Census [26]. The city has two zip codes and is 26 square miles with a population density of 817 per square mile. The nearest metropolitan city with a population of over 150,000 is located 38 miles away. The median yearly income of Albertville is lower than Alabama as a whole ($35,878 vs. 40,489). The Hispanic population is concentrated to approximately 17% of the households in the city (Table 1).

**Table 1:** Population and household characteristics in Albertville city

	Population	Households
Total	20,883	7401
Hispanic	5861 (28%)	1229 (17%)
Non-Hispanic	15,022 (72%)	6172 (83%)

Data was collected from participants interviewed by trained research interviewers in door-to-door canvas between June and December 2013. To be included, participants had to be at least 19 years of age, not pregnant, speak English or Spanish fluently, and self-identify as non-Hispanic white or as Hispanic/Latino. Participants were compensated with a gift card for their time. All study procedures were reviewed and approved by the University of Alabama at Birmingham’s Institutional Review Board.

### Area stratified random sampling for recruitment

The goal to recruit an equal number of Hispanic and non-Hispanic participants would have been difficult to achieve by employing a completely random sampling procedure across the entire city. Therefore, a stratified random sampling procedure was created based on the Center for Disease Control and Prevention’s (CDC) Community Assessment for Public Health Emergency Responses (CASPER) sampling methodology [27]. The CASPER approach was developed using cross-sectional epidemiological principles and is a form of a community needs assessment that provides a systematic approach to collecting household information on community public health status. The cluster sampling design involves two stages: selecting clusters based on household proportions and then interviewing a set random number of households in each cluster. The CDC recommends using GIS software in the selection of the sampling frame to allow users to select portions (clusters) of geographically defined areas, such as counties or cities. In addition, GIS software provides the ability to easily develop maps for community interviewers based on the selected clusters. For this reason, CASPER provides a toolbox for use in ArcGIS software to facilitate this methodology. Using this approach in our study involved three stages: (1) division of the sampling area into non-overlapping strata based on Hispanic household proportion; (2) random selection of the designated number of Census blocks from each stratum; and (3) random selection of the designated number of housing units (i.e., survey participants) from each Census block.

#### Stage 1: Divide the sampling area into non-overlapping strata based on Hispanic household proportion

**Fig. 1:**
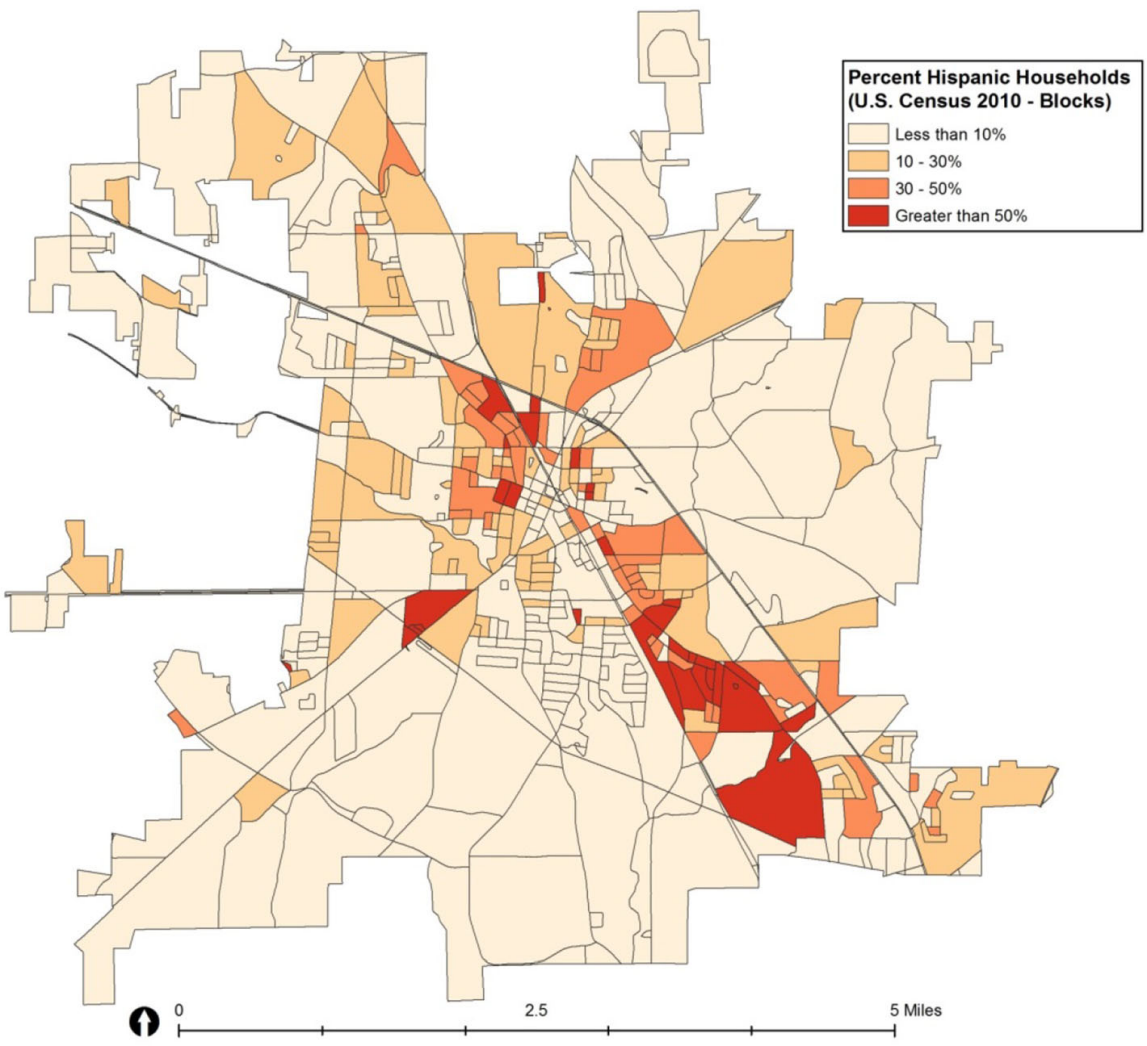
Census blocks in Albertville, AL by Hispanic household proportion. Map of census block groups in Albertville, AL. Darker shading indicates higher Hispanic household proportions. Map developed using licensed ArcGIS software

To ensure that the interviewers would be able to reach sufficient Hispanic households, all Census blocks within Albertville were divided into four strata based on percentage of Hispanic households using GIS software. Since Albertville city boundaries and Census block boundaries do not perfectly align with each other, a centroid criterion was used to determine whether or not a Census block belonged to Albertville city. As a result, 647 Census blocks were assigned to Albertville city. Of those, only 455 blocks contained households and the other 192 blocks were non-residential. Since the Hispanic population was concentrated in a relatively small geographic area, the 455 blocks were further divided into four unbalanced strata identified by Hispanic household proportion: < 10% Hispanic households, 10–30% Hispanic, 30–50% Hispanic, and ≥ 50% Hispanic. Roughly 60% of the blocks were assigned to the ≤10% of Hispanic households stratum, with 7% (*N* = 32) of the blocks assigned to the > 50% of Hispanic households stratum (see Table 2 and Fig. 1).

**Table 2:** Proposed stratified sampling based on Hispanic household proportion

Strata	% Hispanic Households	Eligible Blocks	Selected Blocks	Surveys per Block	Surveys per stratum ^a^	Surveys per Stratum by Hispanic Origin ^b^	
						Hispanic	Non-Hispanic
Stratum 1	≤10%	268	10	6	60	5	55
Stratum 2	10–30%	101	10	6	60	20	40
Stratum 3	30–50%	54	12	4	48	36	12
Stratum 4	> 50%	32	12	4	48	48	0
**Total**		**455**^c^	**44**		**216**	**109**	**107**

^a^Derived by multiplying selected blocks and surveys per block

^b^For strata 1 and 2, distribution of Hispanic and non-Hispanic surveys within each block roughly equate to household proportions. Oversampling of Hispanics was planned a priori to reach recruitment goals thus proportions of Hispanic surveys in strata 3 and 4 were set higher than actual Hispanic household proportions

^c^Total of 647 blocks in Albertvile, AL with 192 blocks with zero household, leaving *n* = 455 eligible blocks for sampling

#### Stage 2: Randomly select the designated number of Census blocks from each stratum

Our goal was to recruit a total of 200 participants, with a distribution of 50% Hispanic and 50% non-Hispanic white (1:1 ratio). Maps denoted that the Hispanic population was largely concentrated in small area blocks (Fig. 1). Although smaller blocks suggest higher population density, they also contain fewer individuals and households compared with larger blocks. Since Hispanics comprised a smaller proportion of total households (17%), we needed to oversample blocks with higher concentrations of Hispanic households in order to reach an equal number of Hispanic and Non-Hispanic surveys. For these reasons we took the following approach to determine the number of Census blocks to select from each group, and the number of housing units to select from each Census block.

1. Considering the varying population size across blocks, it was determined to be more feasible to plan fewer surveys per block in more Hispanic population concentrated areas (i.e., strata 3 & 4 in Table 2), and more surveys per block in more non-Hispanic population concentrated areas (i.e., strata 1 & 2 in Table 2). As a result, we selected 10 blocks with 6 surveys per block from strata 1 and 2 and 12 blocks with 4 surveys per block from strata 3 and 4. These numbers were somewhat arbitrary, balancing the concern that selecting too many blocks which would increase cost, while taking care to not plan for an unrealistic quota of surveys per block when not feasible (e.g. the smallest block in the study area contained only 8 households).
2. For strata 1 and 2, distribution of Hispanic versus non-Hispanic surveys within each block roughly reflected the proportions of Hispanic and non-Hispanic households in the corresponding group. Since oversampling of the Hispanic population was needed to achieve the recruitment goal, proportions of Hispanic surveys in strata 3 and 4 were set higher than the actual proportions of Hispanic households. Table 2 shows the proposed number of blocks to select from each group and numbers of Hispanic versus non-Hispanic surveys projected within each block. In total, we proposed 109 Hispanic surveys and 107 non-Hispanic surveys from 44 blocks.

**Fig. 2:**
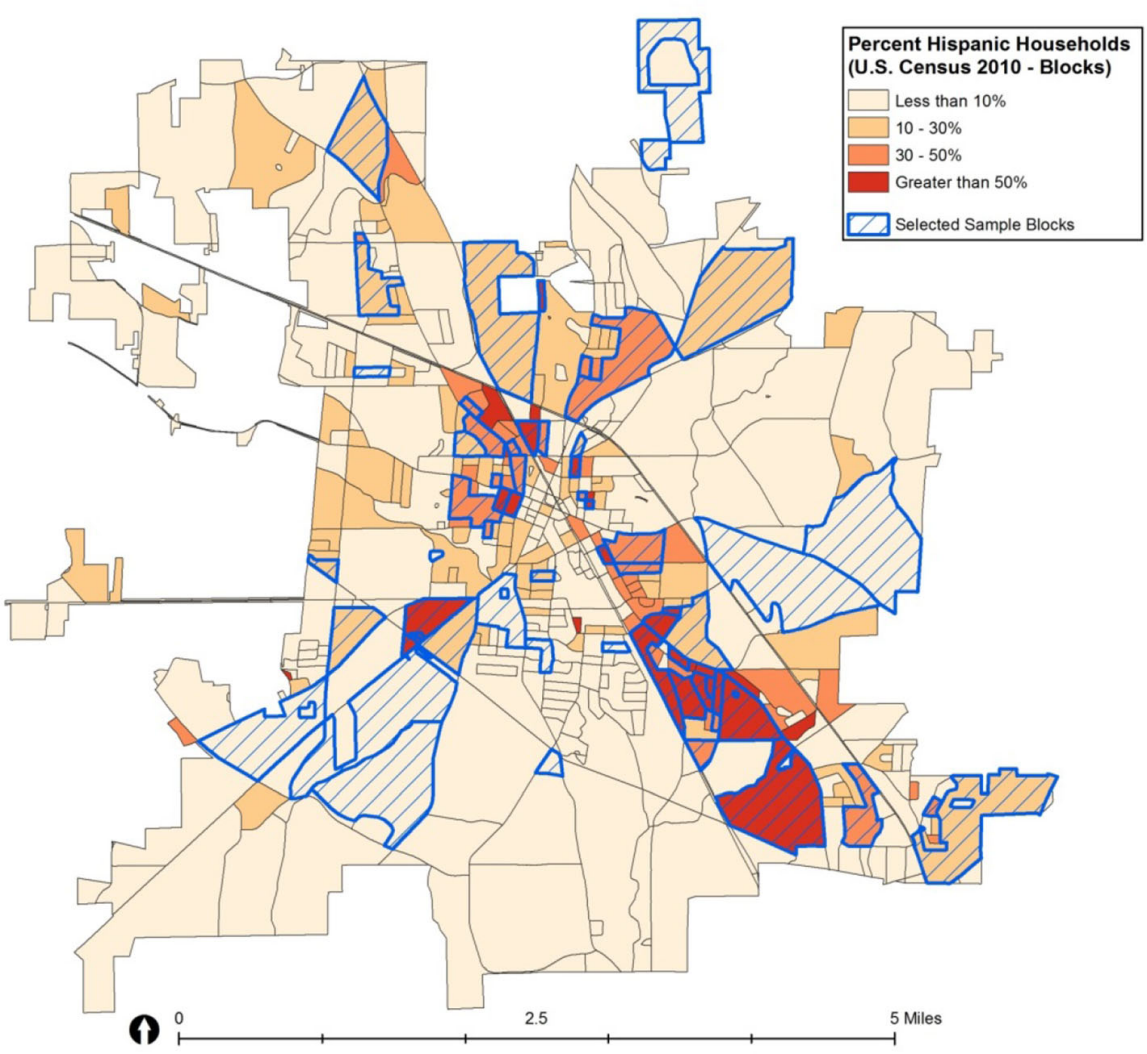
Census blocks selected for recruitment. Map of the 44 census block groups randomly selected in Albertville, AL using an area stratified random sampling approach. Blue outline indicates block group selected. Map developed using licensed ArcGIS software

Once the number of blocks from each group were determined, the CASPER toolkit developed by the CDC was utilized to generate random samples [27]. We used an add-on program developed for ArcGIS by the CDC to generate random samples using a polygon layer that represents the sampling area and non-overlapping clusters within the sampling area. In our study, the four strata were our sampling areas with Census blocks the non-overlapping clusters, accounting for the number of housing units within each cluster. The random sampling procedure was repeated four times, once for each stratum. Figure 2 shows the 44 random blocks selected from the entire study area using this approach.

#### Stage 3: Randomly select the designated number of housing units from each Census block

**Fig. 3:**
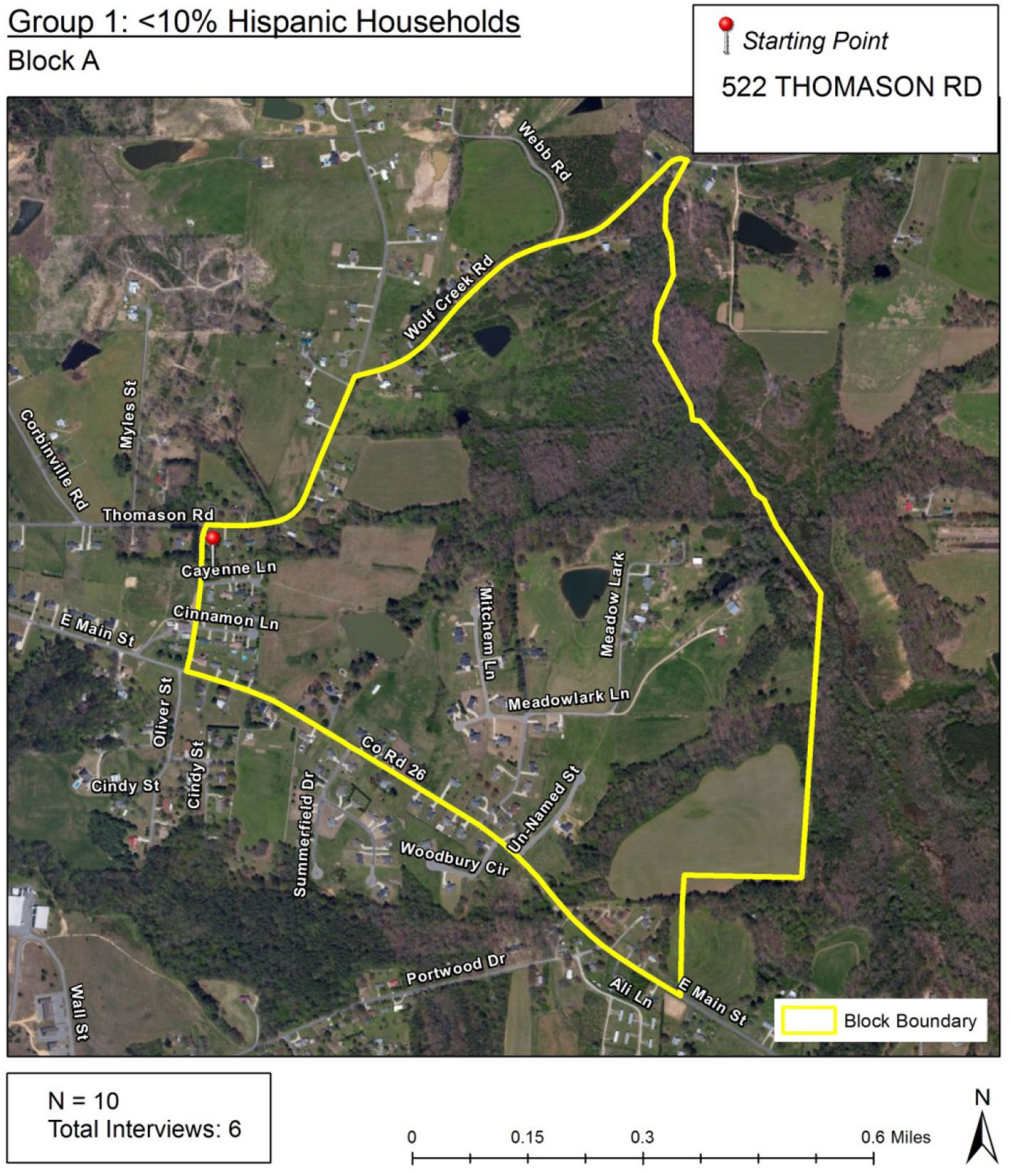
Field interviewer block map. An example of the satellite image map provided to interviewers to conduct field surveys. Map data image provided by© 2013 Google; Imagery© 2013 MaxarTechnologies

Interviewers were provided with satellite maps (Fig. 3) for each block randomized with detailed instructions regarding how to randomly select the designated number of housing units within each block. The systematic random sampling method described in the CASPER toolkit [27] was adapted and modified to develop the study’s survey protocol:

1. A starting point (address) for each sampling block was provided. This was the first house for the interviewers to survey.
2. After completing the first survey, interviewers would walk or drive in either direction to the next N^th^ house. This would be the next household for the interviewers to survey.
3. If no one answers the door, continue to the next N^th^ house.
4. Continue traveling through the sampling block, selecting every N^th^ house until they have completed the designated number of surveys for that sampling block.
5. If the interviewers circled back to the starting point and had not completed the designated number of surveys, they would then proceed through the block again and select every (N + 1)^th^ house. For example, if Block A had an N of 8, in the next pass the interviewer would approach every 9th house.

The N used in the protocol was determined by dividing the total number of housing units by the designated number of surveys to complete in each block, and thus could vary from block to block. For example, if a block contained 50 housing units and the designated number of surveys was 6 for that block, the N would be 8. Values of N for each individual block were provided in the instructions to the interviewers. Additional instructions with regards to abandoned homes, businesses, duplexes and apartment complexes, multiple family homes, and trailer parks were also provided.

## Results

**Fig. 4:**
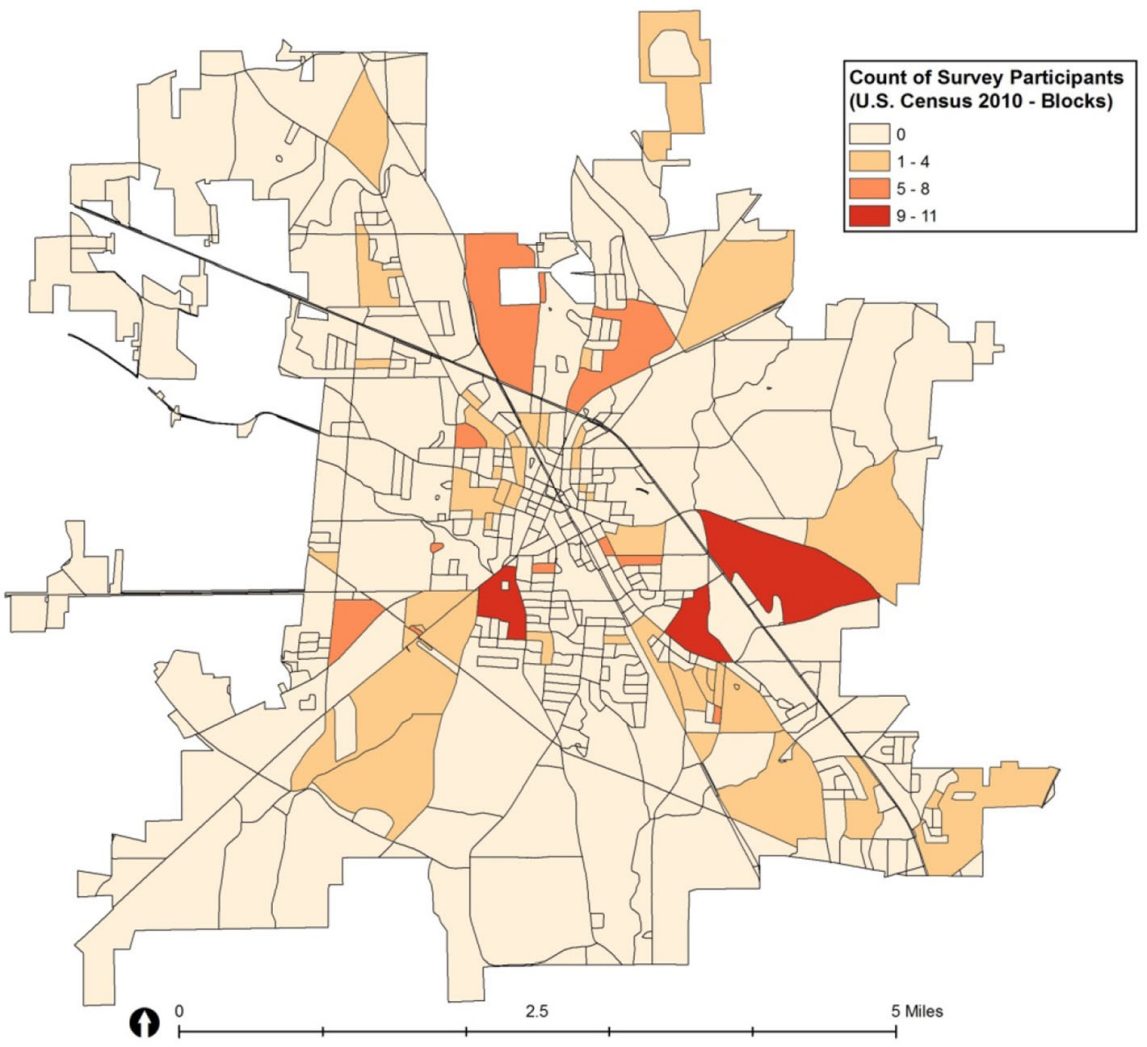
Number of participants by Census block. Map of census block groups in Albertville, AL with the number of participants who completed a survey. Darker shading indicates more participants. Map developed using licensed ArcGIS software

The proposed sample included 109 Hispanic and 107 non-Hispanic participants to be recruited from 44 Census blocks. After exhausting all 44 blocks, interviewers were unable to meet recruitment goals for the proposed number of surveys in each block. Twenty additional blocks were selected using the same random sampling procedure described above, including two from strata 1 (≤10% of Hispanic households), two from strata 2 (10–30% of Hispanic households), six from strata 3 (30–50% of Hispanic households), and ten from strata 4 (> 50% of Hispanic households). More blocks with higher Hispanic population density were selected because field interviewers found that recruitment of Hispanic participants was particularly challenging. The final sample included 106 Hispanic and 111 non-Hispanic participants. The number of surveys completed from each block ranged from 0 to 11, with an average of 3.4 surveys per block (Table 3 and Fig. 4).

**Table 3:** Summary of actual sample

Strata	Proposed Sample				Actual Sample					P ^a^
	Selected Blocks	Hispanic	Non-Hispanic	Group N	Selected Blocks	Hispanic	Non-Hispanic	Group N	% Hispanic Recruited	
Stratum 1 (≤10%) ^b^	10	5	55	60	12	3	41	44	7%	1.0^d^
Stratum 2 (10–30%)	10	20	40	60	12	15	35	50	30%	0.71^c^
Stratum 3 (30–50%)	12	36	12	48	18	33	24	57	58%	0.07^c^
Stratum 4 (> 50%)	12	48	0	48	22	55	11	66	83%	0.002^d^
**Total**	**44**	**109**	**107**	**216**	**64**	**106**	**111**	**217**		

^a^Comparing the proposed distribution of surveys by ethnicity status to the proportions of surveys completed. *P*-values > 0.05 indicate that actual proportions did not differ from proposed population based proportions

^b^Proportion of Hispanic households in each individual block

^c^Chi-square test

^d^Fishers exact test

Post-hoc chi-square and Fishers exact tests were used to test the proposed distribution of surveys by ethnicity status to the proportions of surveys completed. *P*-values > 0.05 indicate that actual proportions did not differ from proposed population based proportions. The proportion of Hispanic surveys completed per strata were similar to our proposed distribution for strata 1–3: 7% for strata 1 (*p* = 1.0), 30% for strata 2 (*p* = 0.71), and 58% for strata 3 (*p* = 0.07). Although Strata 4 (83% Hispanic surveys, *p* = 0.002) had statistically different proportions, this was expected due to the need to oversample Hispanic surveys from this strata.

## Discussion

Here we demonstrate the successful use of a novel area stratified random sampling technique utilizing GIS that ensured ethnic balance in the recruitment of our community canvased study sample. Field recruitment in community studies presents challenges in minimizing selection bias and ensuring demographic representation. Here, integrating GIS based technology with census data provided a standardized and objective approach to recruitment to address these issues. Specifically, we utilized GIS to create and visualize non-overlapping strata to determine individual stratums and to randomly select Census blocks within those strata. Our approach ensured the 1:1 ratio of Hispanics to non-Hispanics in our study, minimized selection bias, and provided an approach that was easy for the ‘boots on the ground’ interviewers to implement. Moreover, the distribution of completed Hispanic surveys by stratum closely matched our original proposed proportions (defined based on percentage of Hispanic households in block), giving our sample geographic representation by Albertville block.

Utilizing GIS to facilitate community-based research, such as targeting areas for program planning or ensuring random sampling of survey respondents [28], has been implemented in recent population based studies. This method has been particularly useful in rural, developing countries [20–22, 29]. Defar et al. used GIS methods to conduct a cross-sectional survey in Ethiopia on maternal and child health care utilization in a similar two-stage process as the current study [29] while Wampler et al. used GIS to facilitate the random selection of households in specific areas in Haiti for water quality research [22]. Akin to the results here, a study that compared simple random sampling to stratified sampling by zip code and census tract found that area based stratified sampling ensured a higher representativeness of Hispanic residents in audits of tobacco retailers in an urban area [30]. In the public health realm, Lafontaine et al. developed a spatial random sampling method to conduct neighborhood built environment audits and concluded that this approach was more cost and time effective [31]. Likewise, using the approach herein resulted in recruiting our Hispanic sample in a more efficient manner.

It is important to note that we selected the number of blocks for randomization and recruitment based on feasibility but nonetheless in an arbitrary fashion. While this resulted in a balanced sample for our study, this will likely not translate into other scenarios. Since stratification by design results in subgroups that are over or under represented compared to the overall population [15], taking the actual population weights of each census tract into account when selecting blocks would have been more appropriate. Since the ultimate goal in sampling is to select a study sample that is representative of the population, applying population sampling weights and using model-based approaches such as raking prior to analysis are essential. Raking adjusts the sampling weights by forcing the survey totals to match proportions in the known population [32].

Our approach was not without challenges or limitations. When conducting the door to door surveys, interviewers were provided with detailed protocol and satellite maps. However, multiple issues arose. First, there was a significant number of houses that provided “no answer” and we had to implement the N + 1 sampling multiple times to reach recruitment targets. Time constraints also impacted interviewers. Some blocks sampled had a count number that was large (*N* > 14), which decreased sampling efficiency as driving from one house to the next could exceed 10 min. Another limitation of the study is that we used the population and household counts from the 2010 Decennial Census data, which may have underestimated the number of Hispanics in Albertville at the time of data collection (2013). Further, the criterion used to divide the study area was Census block group and 2010 Census estimates were likely different than the true distribution of Hispanic households by block in 2013. Lastly, it is important to note that CASPER was designed for use in the United States and associated territories and uses data collected from the census bureau to create population based sampling areas and clusters. However, since CASPER was developed based on an epidemiological two-stage cluster sampling approach, it is possible to conduct this type of sampling in other countries where census type data are available using the CASPER protocol as a guide.

## Conclusion

Overall, we developed a standardized area based randomized sampling protocol that allowed us to successful recruit an ethnically balanced sample while conducting door to door community surveys. Minimizing selection bias in community-based surveys can be difficult; however, advancement in technological tools such as GIS provides novel approaches to address these biases. Based on our results here, we advocate the integration of area based randomized sampling in future community-based research, particularly when trying to reach disparate populations.

## Data Availability

Data sharing is not applicable to this article as no datasets were generated or analyzed during the current study.

## Abbreviations

GIS: Geographic information systems
CASPER: Community Assessment for Public Health Emergency Response
CDC: Centers for Disease Control and Prevention
ACS: American Community Survey
NHANES: National Health and Nutrition Examination Study
